# Direct Hemi-Hypoglossal Nerve and Hypoglossal Nerve for Suprascapular Nerve/Proximal Brachial Plexus Neurotization: A Cadaveric Feasibility Study

**DOI:** 10.7759/cureus.36472

**Published:** 2023-03-21

**Authors:** Katherine Dougherty, Juan J Cardona, Arada Chaiyamoon, Joe Iwanaga, Athikhun Suwannakhan, Erin P. McCormack, Joshua Hanna, Abuzer Güngör, Aaron S Dumont, R. Shane Tubbs

**Affiliations:** 1 Department of Medicine, Tulane University School of Medicine, New Orleans, USA; 2 Department of Neurosurgery, Tulane University School of Medicine, New Orleans, USA; 3 Department of Anatomy, Faculty of Medicine, Khon Kaen University, Khon Kaen, THA; 4 Department of Neurology, Tulane University School of Medicine, New Orleans, USA; 5 Department of Anatomy, Faculty of Science, Mahidol University, Bangkok, THA; 6 Department of Anatomy, Faculty of Science, In Silico and Clinical Anatomy Research Group (iSCAN) Mahidol University, Bangkok, THA; 7 Department of Neurosurgery, Ochsner Neuroscience Institute, Ochsner Health System, New Orleans, USA; 8 Department of Neurosurgery, Yeditepe University School of Medicine, Istanbul, TUR; 9 Department of Neurosurgery, Bakırköy Research and Training Hospital for Psychiatry, Neurology and Neurosurgery, Istanbul, TUR; 10 Department of Structural and Cellular Biology, Tulane University School of Medicine, New Orleans, USA; 11 Department of Surgery, Tulane University School of Medicine, New Orleans, USA; 12 Department of Anatomical Sciences, St. George’s University, St. George’s, GRD

**Keywords:** neurotization, tongue, surgery, upper limb, anatomy

## Abstract

Introduction

Partial restoration of shoulder function is important in upper brachial plexus lesions, and the suprascapular nerve is often the target for such neurotization procedures. Although there is an extensive history of peripheral nerve surgeons using the hypoglossal nerve for various local nerve transfers, some have reported using this nerve as a donor for upper brachial plexus grafting procedures. We discuss our anatomical findings for the use of a direct hypoglossal to suprascapular nerve transfer.

Materials and methods

Fifteen adult cadavers (30 separate sides) were dissected to reveal the hypoglossal nerve in the neck and the supraclavicular brachial plexus in the supraclavicular region. On 15 sides, the hypoglossal nerve was dissected anteriorly to the midline, cut, and transposed toward the supraclavicular region in half of the dissections. On the remaining sides, the nerve was hemisected longitudinally into two equal parts, and the cut inferior portion also swung inferiorly toward the supraclavicular region. The cut end of the hypoglossal nerve was brought toward the proximal suprascapular nerve, the fifth (C5) and sixth (C6) cervical nerve roots, and the upper trunk. Measurements included the length and diameter of the cervical portion of the hypoglossal nerve and the diameter of the suprascapular nerve.

Results

The mean diameter and length of the hypoglossal nerve were 2.1 millimeters (mm) and 72.8 mm, respectively. The mean diameter of the proximal suprascapular nerve was 2.7 mm. Successful, tension-free transposition to the C5 and C6 nerve roots was achieved on all sides. The average extra length of the hypoglossal nerve for a C5 root transposition was 8 mm and 5.2mm for a C6 root transposition. The distal hypoglossal nerve reached the upper trunk on all but two sides (6.7%). The distal hypoglossal nerve reached the proximal suprascapular nerve on all but four sides (13.3%). Of the 87% of sides (n=26) where the hypoglossal nerve reached the proximal suprascapular nerve, 58% of these (n=15) required some manipulation of the suprascapular nerve from its origin at the upper trunk. This technique resulted in a mean additional length to the suprascapular nerve of 35 mm. No differences were found between the completely cut hypoglossal nerves and hemisected nerves in regard to working length.

Conclusions

To our knowledge, the use of the hypoglossal nerve as a transpositional graft for direct suprascapular nerve neurotization has not been previously described. Based on our study, we propose that the hypoglossal nerve, or hemi-hypoglossal nerve, should be considered as a donor nerve to restore suprascapular nerve function in the majority of patients. Additionally, the hypoglossal nerve may be transferred to the C5 and C6 roots and upper trunk of the brachial plexus for direct neurotization.

## Introduction

The hypoglossal nerve supplies all of the muscles of the tongue, except the palatoglossus, which is innervated by the pharyngeal plexus. The rootlets of the hypoglossal nerve derive from the medulla oblongata, then run posterolateral to the vertebral artery to converge within the hypoglossal canal, and exit the skull base as a single nerve. The hypoglossal nerve descends to enter the superior portion of the carotid sheath, medial to the internal jugular vein and lateral to the external carotid artery and vagus nerve. At the angle of the mandible, it crosses anterior to the vagus nerve and then courses along the inferior edge of the occipital artery. Deep to the posterior belly of the digastric muscle, the deeper hypoglossal nerve travels between the deeper hyoglossus muscle and the more superficial mylohyoid muscle. Superior to the greater horn of the hyoid bone, the nerve travels beyond the hyoglossus muscle and deep to the stylohyoid to pass the lateral border of the genioglossus where it terminates near the tip of the tongue [1,2].

The use of the hypoglossal nerve as a nerve graft is well established, most commonly for reinnervation of the facial nerve to restore facial muscle function. This technique was first described by Körte [3] in 1903 who connected the stump of the facial nerve side-to-side to the hypoglossal nerve, without an interpositional graft. This procedure was successful in restoring voluntary facial movement without major long-term deficits to tongue function. Thirty years later, Ballance and Duel [4] proposed a direct end-to-end nerve repair by complete transection of the hypoglossal nerve; however, this technique resulted in tongue atrophy that can impact speech and swallowing. The hemi-hypoglossal-facial nerve transfer was proposed in the 1990s and boasted successful restoration of facial movement while minimizing morbidity [5]. Variations of this technique have since been refined over the past 30 years with favorable results [6-10].

The hypoglossal nerve transfer has rarely been used for the repair of brachial plexus injuries as it traditionally required the use of interpositional nerve grafts, often the sural nerve [11-14]. However, due to the established success with hypoglossal-facial nerve grafts for the restoration of voluntary muscle function, we hypothesized that direct hypoglossal-suprascapular nerve transfers may be possible. As cadaveric feasibility studies can reach patient care faster than traditional translational research, our aim is to provide a potential new surgical technique for patients with lesions of the suprascapular nerve [15].

## Materials and methods

**Figure 1:**
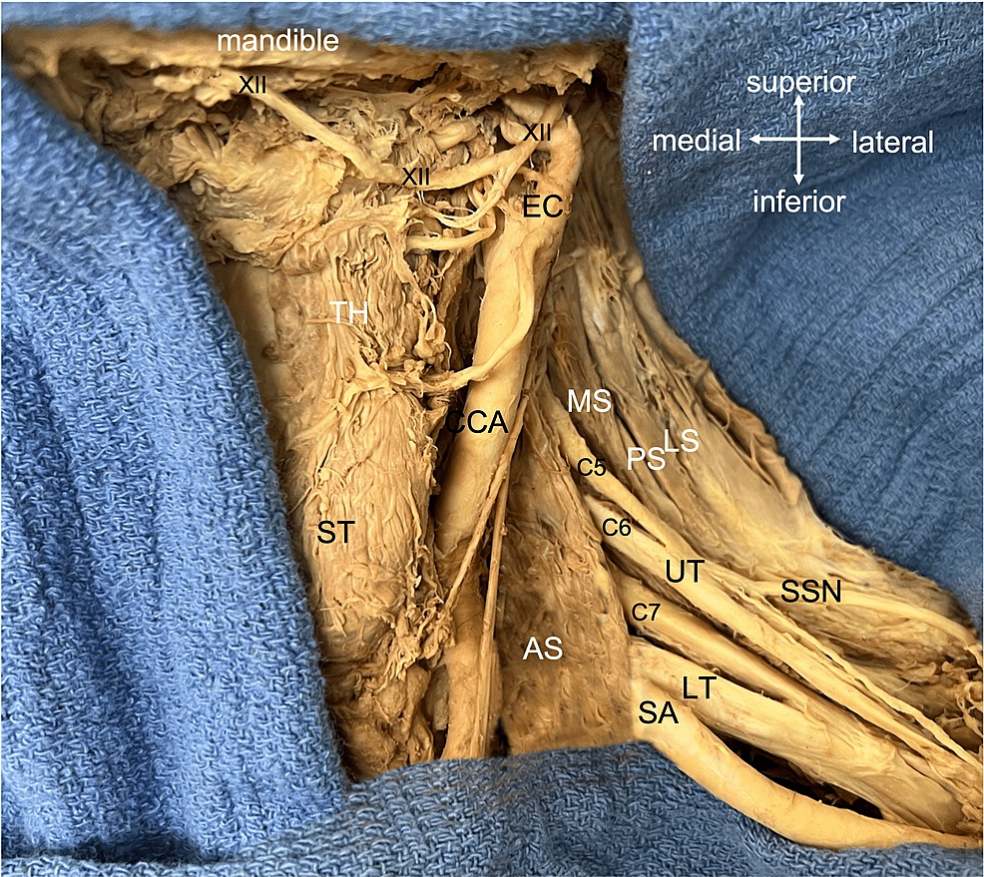
Left neck dissection specifically noting the hypoglossal nerve (XII) and proximal brachial plexus. The ventral rami of C5-C7 are shown along with the UT and LT of the brachial plexus. The SSN is shown, as well as the SA, AS, MS, and PS muscles, LS, CCA, ST, and TH muscles, and EC. C5: fifth cervical nerve root, C7: seventh cervical nerve root, UT: upper trunk, LT: lower trunk, SSN: suprascapular nerve, SA: subclavian artery, AS: anterior scalene, MS: middle scalene, PS: posterior scalene, LS: levator scapulae, CCA: common carotid artery, ST: sternothyroid, TH: thyrohyoid, EC: external carotid artery

In the supine position, 15 adult cadavers (30 separate sides) (75% confidence level, 10% margin of error, and 50% population proportion) underwent dissection of the cervical hypoglossal nerve and the supraclavicular brachial plexus (Figure 1).

The specimens were lightly fixed in formalin and included eight female and seven male specimens with a mean age at death of 78 years (range: 58-88 years). With the head slightly rotated contralaterally, the hypoglossal nerve was identified deep to the posterior belly of the digastric muscle, and it was dissected anteriorly, tracking its course between the more superficial mylohyoid muscle and the deeper hyoglossus muscle. The mylohyoid muscle was transected, and the nerve followed to the midline. The sternocleidomastoid was removed from its clavicular and sternal attachments and retracted superolaterally. On 15 sides, at the midline, the nerve was cut and reflected inferiorly toward the supraclavicular region. On the remaining 15 sides, the nerve was hemisected longitudinally into two equal parts, and the cut inferior portion was also swung inferiorly toward the supraclavicular region (i.e., hemi-hypoglossal nerve transfer). Next, the fifth (C5) and sixth (C6) cervical roots, the upper trunk, and the proximal suprascapular nerve were identified. The cut end of the hypoglossal nerve was brought toward these aforementioned nerves, and the anatomical associations between these structures were accessed. Excess nerve following after transposition to each of these nerves was measured. When necessary, for a direct tension-free neurotization with the hypoglossal nerve, the proximal suprascapular nerve was teased away from its origin as previously described [16].

Measurements included the length and diameter of the cervical portion of the hypoglossal nerve and the diameter of the suprascapular nerve. Specifically, the length of the hypoglossal nerve was measured from its cut portion up to the angle of the mandible termed its working length. All measurements were made with microcalipers (Mitutoyo, Japan) and by two observers (KD and RST). All measurements were made three times, and the average was taken. Statistical analysis was performed with statistical significance set at p<0.05 (Wizard for Mac, Evan Miller) (https://www.wizardmac.com/). Every effort was made to follow all local and international ethical guidelines and laws that pertain to the use of human cadaveric donors in anatomical research [17].

## Results

**Figure 2:**
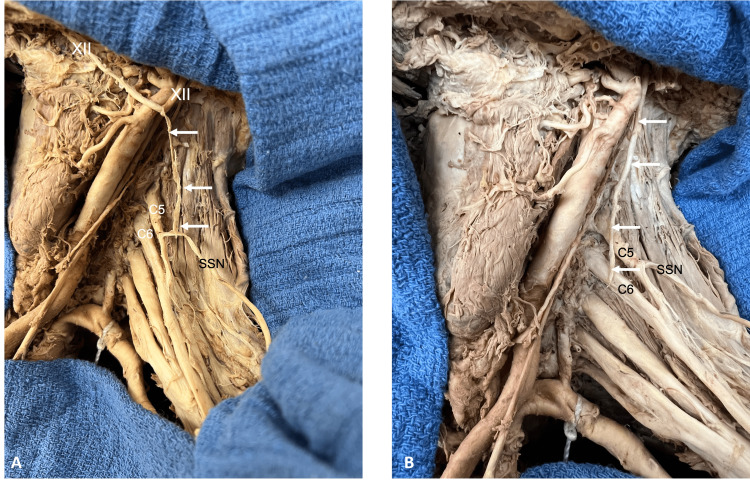
Left neck dissections. A: Hemi-hypoglossal nerve (XII) dissection with the inferior part of the nerve (arrows) being transposed to the proximal SSN. Also, note the proximity to the C5 and C6 ventral rami. B: Entire hypoglossal nerve (arrows) being transposed to the C6 ventral ramus. Also, note the SSN. SSN: suprascapular nerve, C5: fifth cervical root, C6: sixth cervical root

The mean diameter of the hypoglossal nerve was 2.1 millimeters (mm) (range: 1.3-3.26 mm), and the mean length was 72.8 mm (range: 57-91 mm). After cutting the hypoglossal nerve at the midline and reflecting the entire nerve inferiorly toward the supraclavicular brachial plexus, this nerve reached the C5 and C6 roots, tension-free, on all sides (Figure 2).

**Figure 3:**
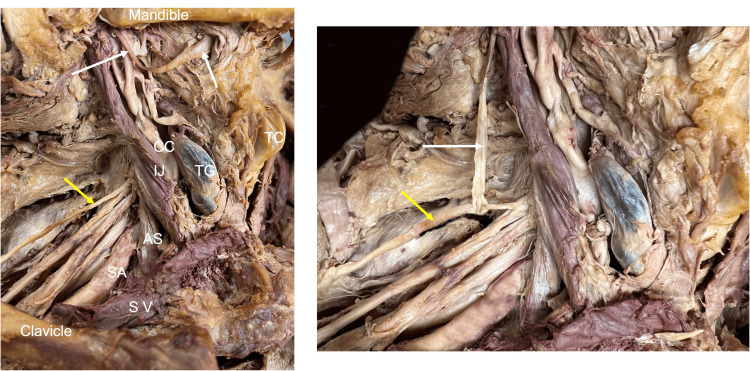
Right side neck dissection illustrating the regional anatomy (left) of the hypoglossal nerve (white arrows) and suprascapular nerve (yellow arrows) and then the complete transposition of the hypoglossal nerve to the suprascapular nerve (right). White arrows indicate the hypoglossal nerve, and yellow arrows indicate the suprascapular nerve. CC: common carotid artery, IJ: internal jugular vein, TC: thyroid cartilage, TG: thyroid gland, AS: anterior scalene, SA: subclavian artery, SV: subclavian vein

**Figure 4:**
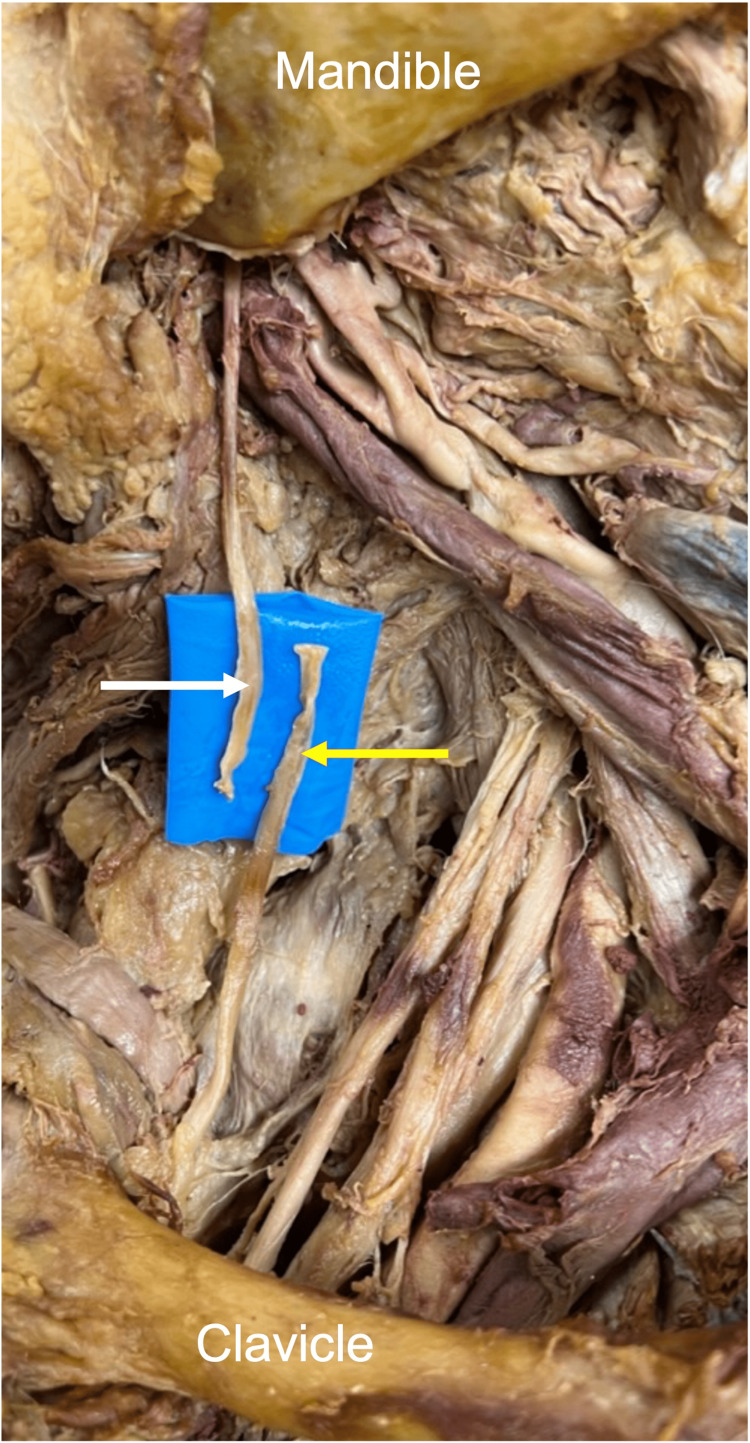
Right side dissection seen in Figure 3 showing the excess nerve length for complete hypoglossal nerve (white arrow) transposition to the transected proximal end of the suprascapular nerve (yellow arrow).

The average excess length of the hypoglossal nerve with a C5 root transposition was 8 mm (range: 7-13 mm). The average excess length of the hypoglossal nerve with a C6 root transposition was 5.2 mm (range: 4-9 mm). The distal hypoglossal nerve reached the upper trunk on all but two sides (6.7%). The distal hypoglossal nerve easily reached the proximal suprascapular nerve on all but four sides (13.3%). Of the 87% of sides (n=26) where the hypoglossal nerve reached the proximal suprascapular nerve, 58% of these (n=15) required some manipulation of the suprascapular nerve from its origin at the upper trunk of the brachial plexus, as previously described (Figure 3) [17]. This technique resulted in a mean additional length of 35 mm (range: 20-44 mm) (Figure 4).

The mean diameter of the proximal suprascapular nerve was 2.7 mm (range: 2.5-2.91 mm). No statistically significant differences were found between sex or when comparing the left and right sides. No statistically significant differences were found between completely cut hypoglossal nerves and hemisected nerves, although it was felt that there was usually a more tension-free transposition when using the completely cut hypoglossal nerves (Figure 2). No specimen was found to have signs of previous surgery to the areas dissected.

## Discussion

The suprascapular nerve as it courses through the suprascapular and spinoglenoid notches renders it susceptible to trauma, anatomical entrapment, or impingement [18,19]. The suprascapular nerve provides motor innervation to the supraspinatus and infraspinatus muscles, which are essential for abduction and lateral rotation of the arm. We found that the hypoglossal or hemi-hypoglossal nerve was suitable for direct transfer to the proximal suprascapular nerve on the majority of sides. The hypoglossal nerve also reached the C5 and C6 roots, tension-free, on all sides.

Historically, nerve transfers used to reinnervate the suprascapular nerve have included the spinal accessory nerve, phrenic nerve, motor branches of the C4 root, and hypoglossal nerve [5,18,19]. Although less common, there has been success utilizing the hypoglossal nerve in some brachial plexus injuries [12,20,21]. In a retrospective study by Malessy et al. [22], hypoglossal nerve transfers were used in combination with other nerve grafts in eight patients to restore shoulder function. Reinnervation of the supraspinatus was achieved in all cases, and infraspinatus reinnervation was achieved in 75% of cases [22]. Anastakis et al. [12] used the hypoglossal nerve for neurotization procedures to the axillary, median, lateral pectoral, musculocutaneous, and ulnar nerves. These authors reported minimal donor site morbidity and good functional results [12]. In an anatomical feasibility study by Liao et al. [11], the hypoglossal nerve was considered for reinnervation of the obturator nerve or thoracodorsal nerve in free functional muscle transfers used to restore elbow flexion. Their results showed that the length of the nerve (7-12 cm) was sufficient for transfer without the need for an additional nerve graft [11].

In our previous cadaveric study, it was shown that the suprascapular nerve could be considered a donor nerve for facial nerve reanimation procedures. The mean axonal count for the suprascapular nerve at its origin from the brachial plexus is approximately 6,000 [16]. As the axonal count of the hypoglossal nerve is approximately 9-10,000, it would be sufficient for reinnervation of the suprascapular nerve, even when hemisected as demonstrated clinically by Al-Thunyan et al. [20]. Longitudinal splitting of the hypoglossal nerve was also successful in our specimens and still offers approximately 4,500 axons for transfer. We also determined that the hypoglossal nerve easily reaches the C5 and C6 roots and the upper trunk without any significant tension. As root avulsion is a clinical challenge affecting the upper trunk of the brachial plexus, such a transfer should be considered by the peripheral nerve surgeon. Earlier case reports have shown that such a transfer has good clinical outcomes (e.g., biceps brachii function with the use of interpositional grafts) [20]. To our knowledge, all of these have used interpositional nerve grafts to reach the brachial plexus as the hypoglossal nerve was cut too proximally. We posit that using our midline dissection technique for the hypoglossal nerve will minimize the need for interpositional grafting and may even result in improved clinical outcomes as direct transfers usually yield superior outcomes [20].

Declaration

We sincerely thank those who donated their bodies to science so that anatomical research could be performed. Results from such research can potentially increase mankind’s overall knowledge that can then improve patient care. Therefore, these donors and their families deserve our highest gratitude [23].

## Conclusions

To our knowledge, the use of the hypoglossal nerve as a nerve donor for direct suprascapular nerve neurotization has not been previously explored. Based on our study, we propose that the hypoglossal nerve, or hemi-hypoglossal nerve, should be considered a donor nerve to restore suprascapular nerve function. Additionally, using our dissection methods, the hypoglossal nerve may also be transferred to the C5 and C6 roots and upper trunk of the brachial plexus for direct neurotization.
